# Effects of Micro-Shot Peening on the Fatigue Strength of Anodized 7075-T6 Alloy

**DOI:** 10.3390/ma16031160

**Published:** 2023-01-29

**Authors:** Chih-Hang Su, Tai-Cheng Chen, Yi-Shiun Ding, Guan-Xun Lu, Leu-Wen Tsay

**Affiliations:** 1 Department of Optoelectronics and Materials Technology, National Taiwan Ocean University, Keelung 20224, Taiwan; 2 Nuclear Fuels and Materials Division, Institute of Nuclear Energy Research, Taoyuan 32546, Taiwan; 3 Material Research Group, Asia Development Center, SRAM LLC, Taichung 40765, Taiwan

**Keywords:** AA 7075-T6 alloy, anodizing, micro-shot peening, nanograin, rotating bending fatigue

## Abstract

Micro-shot peening under two Almen intensities was performed to increase the fatigue endurance limit of anodized AA 7075 alloy in T6 condition. Compressive residual stress (CRS) and a nano-grained structure were present in the outermost as-peened layer. Microcracks in the anodized layer obviously abbreviated the fatigue strength/life of the substrate. The endurance limit of the anodized AA 7075 was lowered to less than 200 MPa. By contrast, micro-shot peening increased the endurance limit of the anodized AA 7075 to above that of the substrate (about 300 MPa). Without anodization, the fatigue strength of the high peened (HP) specimen fluctuated; this was the result of high surface roughness of the specimen, as compared to that of the low peened (LP) one. Pickling before anodizing was found to erode the outermost peened layer, which caused a decrease in the positive effect of peening. After anodization, the HP sample had a greater fatigue strength/endurance limit than that of the LP one. The fracture appearance of an anodized fatigued sample showed an observable ring of brittle fracture. Fatigue cracks present in the brittle coating propagated directly into the substrate, significantly damaging the fatigue performance of the anodized sample. The CRS and the nano-grained structure beneath the anodized layer accounted for a noticeable increase in resistance to fatigue failure of the anodized micro-shot peened specimen.

## 1. Introduction

Surface technologies are extensively applied to components in the aerospace, automobile, and power industries to protect against corrosion and wear. Thermal spray coating, electroplating, and chemical and physical vapor deposition are employed to modify the surface characteristics of the components. The most typical approach to increase the wear and corrosion resistance of Al alloys is to coat them with anodic oxide film [1,2]. An oxide film formed on the surface of anodized Al alloys consists of a thin compact inner layer and a porous outer layer [2]. A major concern about the use of AA 7075 Al alloy is the possible fatigue failure of structural components [3,4,5]. The anodization process is reported to obviously degrade the fatigue performance of high-strength Al alloys [6,7,8,9,10,11].

The effect of anodization on the fatigue strength/life of Al alloys is counted on several process variables. Moreover, the fatigue strength of a soft anodized coating is superior to that of a hard anodized one [12]. Chromic acid anodizing is less harmful to fatigue strength than sulfuric acid anodizing [13,14]. Increasing the thickness of the anodizing coating lowers the fatigue strength/life of AA 7075 alloy [10]. Moreover, a coating on an ultrafine-grained substrate has shown enhanced resistance to anodizing-induced and fatigue-induced cracking of AA 6682 alloy [12]. It is reported that the microcracks in the anodized film and irregularities beneath the film are the reasons for the decrease in fatigue strength [10]. The loss in fatigue strength of anodized AA 2219 and AA 2024 alloys is related to the induced microcracks at the etch pits, which are formed from the preferential dissolution of intermetallics in pickling performed before anodizing [7]. In one study, Al_2_O_3_ coatings were applied on the AA 6061-T6 alloy by hard anodizing, micro-arc oxidation, detonation spray, and air plasma spray, and hard anodizing was found to have the most damaging effect on fatigue endurance [15].

Shot peening is employed to upgrade the fatigue performance of the Al alloys [16,17,18]. The resulting compressive residual stress (CRS), strain hardening, and fine-grained microstructure in the shot-peened layer are the main causes of improved fatigue properties. Successful fatigue enhancement depends on a compromise between the CRS and the detrimental effect on surface quality [19]. Advanced shot peening technologies, such as laser [20,21], ultrasonic [22,23], and waterjet peening [24,25], are employed to reduce the irregularity and upgrade the fatigue life of Al alloys. It is noted that shot peening can mitigate the harmful effect of an anodized coating on the fatigue strength of high-strength Al alloys [13,21,26]. With shot peening, the fatigue strength of anodized AA 6082-T651 alloy is even higher than that of the unanodized substrate [26]. In 3.5% NaCl solution, the fatigue lives of AA 7075-T73 alloy can be markedly increased by shot peening treatments as compared to an electropolished sample [27]. To reduce surface irregularity, the use of fine particles for peening [28,29] is more economical and practical than other advanced peening processes.

AA 7075-T6 alloy has been widely used in the aerospace industry, which can be heat-treated to achieve various microstructures and properties [30]. A decrease in the surface roughness of machined AA 7075 alloy can be achieved by increasing the cutting speed, increasing the cutting depth, or decreasing the feeding rate [30]. The aim of this work was to explore the application of micro-shot peening as a pretreatment before anodizing, which provided a means to restore the fatigue performances of anodized AA 7075 alloy. The fatigue limit of the specimens was evaluated by a rotating bending machine at room temperature. The effect of hard anodizing on the fatigue limit/life of the unpeened and peened AA 7075 alloy was investigated. Plastic deformation of the micro-shot peened specimen was measured by using the Almen intensity, and the outer roughness was detected with a 3D contour profiler. The surface morphologies of ruptured specimens after fatigue tests were examined with a scanning electron microscope (SEM). The refined structure in the peened samples was identified by the inverse pole figure (IPF) map.

## 2. Material and Experimental Procedures

### 2.1. Sample Preparation

The fatigue tests were performed on AA 7075 bar with 10 mm diameter. The chemical composition (wt.%) of the alloy bar was as follows: 1.462 Cu, 2.504 Mg, 5.854 Zn, 0.029 Mn, 0.224 Cr, 0.07 Si, 0.117 Fe, 0.023 Ti, and the residual Al. Moreover, AA 7075 plate with a thickness of 8 mm was used for surface metrology examination and residual stress determination of the samples. The composition in wt.% of the plate was as follows: 1.294 Cu, 2.420 Mg, 5.616 Zn, 0.015 Mn, 0.292 Cr, 0.040 Si, 0.192 Fe, 0.020 Ti, and the balance Al. All the samples were solution-annealed at 743 K for 1 h, followed by water-quenching, and then aged at 393 K for 24 h. The solution-annealed and aged AA 7075 alloy was named the base metal (BM) sample. The BM had a yield strength, tensile strength, and tensile elongation of 618 MPa, 668 MPa, and 9%, respectively [31]. The tested specimens were ground with SiC paper before micro-shot peening. Micro-shot peening was performed on the ground samples using amorphous powders with sizes of 50–80 μm under 200% surface coverage. The two micro-shot peen intensities employed in this work were determined from the height of the N-type Almen specimen to be 0.110 mm (low peen, LP) and 0.204 mm (high peen, HP). The micro-shot peened samples were distinguished as LP and HP according to the Almen intensity.

### 2.2. Sulfuric Hard Anodizing

Some of the unpeened and peened samples were subjected to anodizing treatment. Prior to the hard anodization process, the sample surface was thoroughly cleaned by degreasing, acid activation, and chemical polishing to totally remove the surface contaminants. Pickling acts chemically to remove the oxides, inclusions, and compounds from the sample surface. Hard anodizing to a thickness of 30 μm was performed in an electrolyte (20 wt.% sulfuric electrolyte + 5 wt.% aluminum sulfate) at a bath temperature of −3 to 3 °C under a varying voltage (25 to 45 V). Sealing was performed in nickel acetate solution at 85–92 °C. For anodized samples, the symbol A affixed to the specified sample (e.g., HPA denoted the anodized high peened sample).

### 2.3. Hardness Measurement and Fatigue Testing

**Figure 1:**
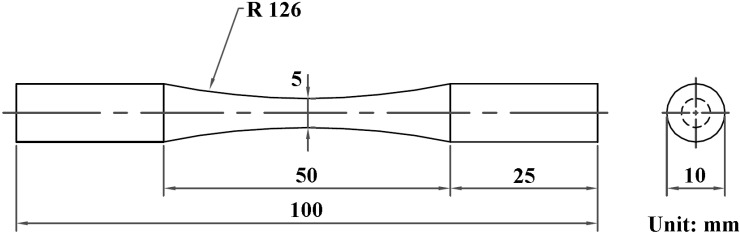
Dimensions of the fatigue test specimen used in this study.

An MVK-G1500 Vickers hardness tester (Mitutoyo, Kawasaki, Japan) was applied to determine the hardness of the samples. The anodized coating was loaded at 10 gf for 15 s. Moreover, a Hysitron TI 980 nanoindenter (Bruker, Billerica, MA, USA) loaded at 2000 μN was used to determine the change in hardness around the interface between the anodized and peened layers. Surface metrology of the samples with and without micro-shot peening was performed with a Contour GT-K 3D optical profiler (Bruker, Billerica, MA, USA), which provided noncontact surface measurements. Figure 1 displays the dimensions of the dog-bone samples for fatigue tests. Rotary bending fatigue tests were conducted at room temperature and a frequency of 33.3 Hz at R = −1 (fully reversed). Fatigue stress (S) vs. number of cycles (N) to failure of the tested samples was measured, and the results presented herein are the averages of three samples, although individual values are also reported.

### 2.4. Microstructural and Fracture Surface Observations

The microstructures of the samples after metallurgical preparation were examined using an S-4800 SEM (Hitachi, Tokyo, Japan). The samples were also detected using an SEM equipped with a NordlysMax^2^ electron backscatter diffraction (EBSD, Oxford Instruments, Abingdon, UK) detector to identify the refined structure of the inspected specimens. Moreover, the strain fields around the interface between the coating and substrate of the peened samples after anodizing were analyzed using the HKL Channel 5 software (Oxford Instruments, Abingdon, UK) to process the original data obtained by the EBSD. The macro-fracture appearance and the detail of the fracture features of the fatigue-fractured specimens were examined using an S-3400N SEM (Hitachi, Tokyo, Japan).

### 2.5. Residual Stress Measurement

The µ-X360s (Pulstec, Hamamatsu, Japan), a residual stress analyzer, was applied to determine the distribution of residual stress in a micro-shot peened sample. The standard settings of the X-ray source were using Cr target Kα radiation (wavelength 2.291 Å) at an X-ray tube voltage of 30 kV with 1.5 mA current. The device for measuring residual stress was based on the cos α method. The full width at half maximum (FWHM) of the (311) peak was related to the distortion of the lattice. The distribution of residual stress in the thickness direction was obtained by removing the surface layer of the sample using an EP-3 electrochemical polisher (Pulstec, Hamamatsu, Japan).

## 3. Results

### 3.1. Micro-Shot Peening and Morphology

**Figure 2:**
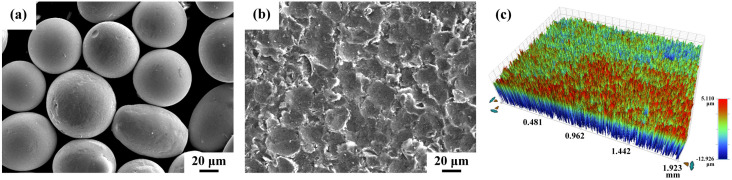
Typical appearance of the (**a**) amorphous pellet, (**b**) surface morphology, and (**c**) surface roughness of the micro-shot peened specimen.

The morphology of the amorphous shot and the surface appearance of the micro-shot peened sample are displayed in Figure 2. The individual pellet of the amorphous shot ranged from 50 to 80 μm in size (Figure 2a). As reported in previous studies [28], the amorphous pellet had a Vickers microhardness of about HV 1150. The surface morphology of the micro-shot peened samples (Figure 2b) had indentations of 10–30 μm in size. AA 7075 BM was ground with SiC paper before micro-shot peening. The surface roughness of the BM and two micro-shot peened samples (LP and HP), determined by a 3D contour profiler, are listed in Table 1. The ground sample had Sa, Sp, and Sv values of 0.217, 1.494, and −1.211 μm, respectively. Continuous bombardments with amorphous shot increased the surface roughness of the peened sample, especially that of the HP sample. The Sa, Sp, and Sv roughness values of the LP one were 0.385, 1.396, and −1.506 μm, accordingly. This revealed that the LP specimen had a slightly higher surface roughness than the unpeened sample. The HP sample had an obviously higher surface roughness than the other two samples; the Sa, Sp, and Sv were 1.303, 6.400, and −7.519 μm, respectively. In general, such a high surface roughness of the HP sample was expected to degrade the resistance to fatigue cracking.

**Figure 3:**
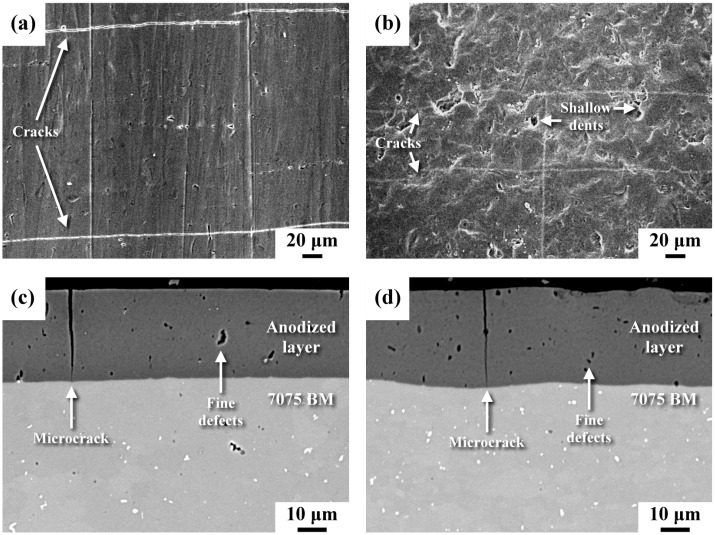
(**a**,**b**) Top view and (**c**,**d**) cross-sectional view of the anodized sample: (**a**,**c**) the unpeened sample; (**b**,**d**) the micro-shot peened sample.

Figure 3 displays the top and cross-sectional views of anodized samples with or without micro-shot peening. The top surface features of the unpeened anodized sample showed regular rectangular cracks (Figure 3a). In the HPA sample, shallow dents with vague cracks were visible on the top surface (Figure 3b). It was obvious that the surface topography and roughness of the anodized samples were affected by the surface texture of the substrate. The cross-sectional views of the anodized layers of different samples are shown in Figure 3c,d, revealing that the thickness of the anodized layer was about 30 μm in both samples. Without peening, the top surface and the interface between the anodized layer and the substrate were quite flat (Figure 3c). Some deep microcracks and fine defects of uneven sizes were present in the anodized layer (Figure 3c). Overall, the anodized peened samples (HPA and LPA) and the BMA had similar microstructures within the anodized zone. The difference between them was that the straight interfaces of the unpeened sample (Figure 3c) were replaced by a slightly tortuous profile of the peened one (Figure 3d). It was found that micro-shot peening did not reduce the number of defects in the anodized zone. Within the anodized zone, the open crack width of the peened sample seemed to be narrower than that of the unpeened sample, but the difference was hard to distinguish.

### 3.2. Hardness Measurements

**Figure 4:**
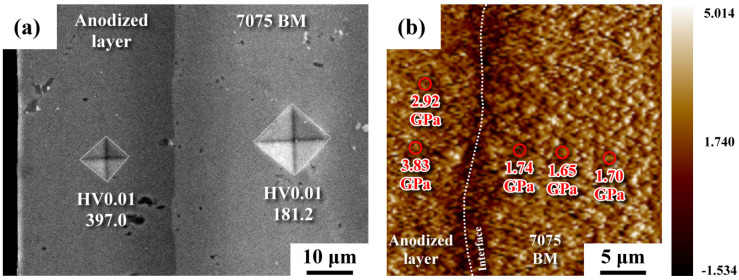
The hardness indentations: (**a**) the micro-Vickers indentations of the anodized sample; (**b**) the nano-hardness distribution around the anodized interface of the micro-shot peened sample.

Figure 4 shows the hardness value of the anodized film and the hardness distribution around the interface between the micro-shot peened zone and anodized layer, as determined by a Vickers hardness tester and nanoindenter, respectively. As shown in Figure 4a, the anodized layer had an apparently higher hardness (around HV 400) than that of the substrate (around HV 180). Thus, the anodized layer improved the resistance to corrosion and wear of the AA 7075 alloy. The hardness indenters were also applied to the specific sites around the interface of the anodized sample. The hardness values determined using a nanoindenter on the right side of the interface (Figure 4b), i.e., the substrate side, were 1.74, 1.65, and 1.70 GPa, respectively. On the other side, hardness values of 3.83 and 2.92 GPa were obtained; these two high values likely belonged to the anodized layer. In prior work [30], the hardness of the micro-shot peened layer was found to exceed 2.94 GPa (HV 300). Thus, the strain hardening and nanocrystalline structure introduced by micro-shot peening could be partly removed by pickling before anodizing.

### 3.3. Microstructural Observations

**Figure 5:**
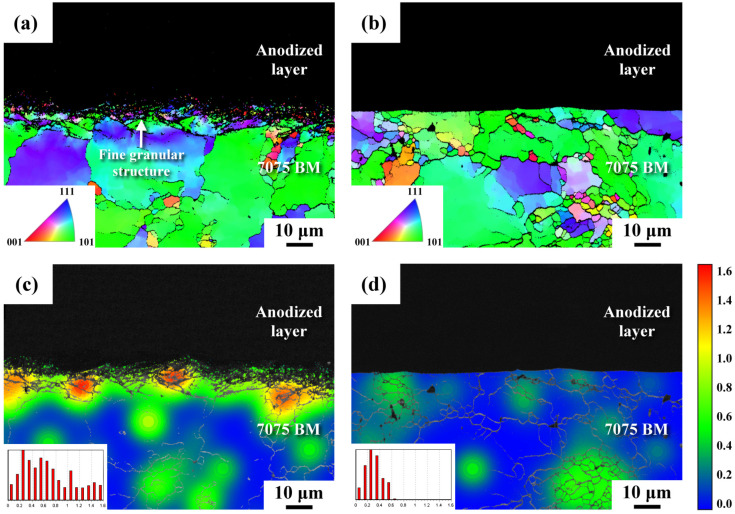
(**a**,**b**) The IPF maps and (**c**,**d**) the strain maps of the inspected samples in cross-sectional view: (**a**,**c**) the HPA sample; (**b**,**d**) the LPA sample.

A highly deformed layer should consist of nanograins in the outermost surface and micron-size elongated grains in the subsurface of the micro-shot peened sample [31]. Figure 5 presents the IPF and strain maps of the HPA and LPA samples, showing the changes in the granular microstructures and strain fields around the interfaces between the substrates and anodized layers. It should be noticed that no Kikuchi pattern was detected within the anodized layer due to its amorphous structure. Because of the limited resolution of the EBSD analysis, the ultrafine grains could not be distinguished and displayed in different colors to show the individual grain boundaries or orientations. The IPF map shows a nanogranular structure present within a depth of 10 μm below the interface of the HPA sample (Figure 5a); this structure was associated with the original micro-shot peened zone. As shown in Figure 5b, no fine granular structure was seen in the LPA sample. The results indicated that only some fine grains occasionally nucleated along the boundaries of coarse grains in the LPA sample. Those refined grains in the micro-shot peened layer were caused by the dynamic recrystallization of the deformed surface layer during the bombardment with fine shot. The original deformed and recrystallized layer disappeared in the LPA sample (Figure 5b), which was attributed to the results of pickling before anodizing. Figure 5c,d show the strain distribution maps, which are in cross-sectional view around the anodizing interfaces of the HPA and LPA specimens. These two figures refer to the IPF maps shown in Figure 5a,b. The high-strain zone is shown in red; the low-strain zone appears in blue. The HPA sample exhibited high strain (in red) within a depth of 20 μm beneath the interface (Figure 5c), which was not seen in the LPA sample (Figure 5d). Those red zones in the HPA sample (Figure 5c) could be related to the severely deformed zones during peening. Moreover, the LPA sample had low strain beneath the interface (Figure 5d). It was confirmed that, in the HPA sample, part of the micro-shot peened zone remained after pickling.

### 3.4. Fatigue Evaluation

**Figure 6:**
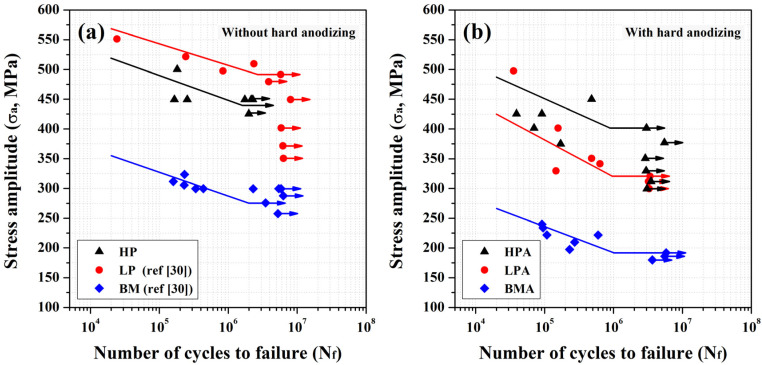
Fatigue stress (S) vs. cycle (N) curves of the samples (**a**) without and (**b**) with a hard anodizing coating.

Figure 6 demonstrates the results of fatigue tests conducted in laboratory air for up to 10^7^ cycles. The fatigue life of the tested samples was sensitive to the loading condition. Without peening and anodizing, the endurance limit of the AA 7075 BM was about 275 MPa [31] (Figure 6a). As revealed in prior work [31], the endurance limit of the LP sample was about 500 MPa, which was much greater than that of the AA 7075 BM (Figure 6a). It was noticed that the fatigue properties of the HP sample were not better than those of the LP specimen (Figure 6a). Furthermore, the HP sample exhibited high fluctuation in fatigue strength/life during testing. The fatigue curves of anodized specimens are shown in Figure 6b. The endurance limit of the anodized specimen (BMA) decreased to about 180 MPa (Figure 6b). It was obvious that hard anodizing had a strongly adverse effect on the fatigue strength of AA 7075 alloy in the high-cycle region. Peening at a low Almen intensity improved the fatigue performance of the LPA sample, as compared with that of the BMA sample (Figure 6b). The endurance limit of the LPA specimen was about 300 MPa, if the BM sample was pretreated with low peening intensity before anodizing. Peening at a high Almen intensity raised the endurance limit of the HPA specimen to about 400 MPa, which was about 100 MPa greater than that of the BM. The results indicated that the peening and pickling strongly influenced the fatigue performance of anodized AA 7075 alloy. Moreover, micro-shot peening could improve the fatigue performance of anodized AA 7075-T6 alloy.

### 3.5. Fractured Surface Examinations

**Figure 7:**
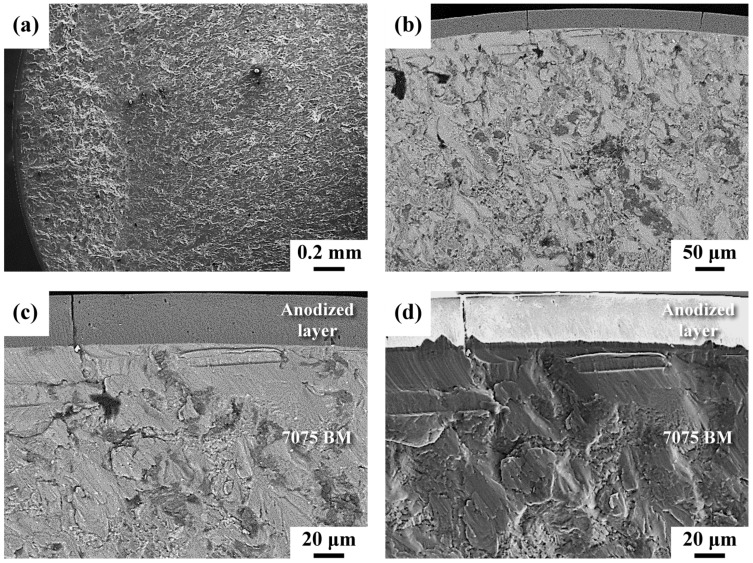
Fatigue fracture appearance of the BMA sample: (**a**,**d**) SE images; (**b**,**c**) BSE images. (**a**) Macro-fractured appearance of the crack initiation site; (**b**) enlarged view of the anodized layer; (**c**,**d**) fracture features around the anodized interface.

The fatigue-fractured morphologies of the anodized BM sample (BMA) are shown in Figure 7. The backscattering electron (BSE) image is more likely to reveal the microcracks and delamination at the interface, whereas the secondary electron (SE) image can show the detailed surface feature of the fatigue-cracked sample. Macroscopically, the main crack grew from the outer surface and into the interior in a radial crack path (Figure 7a). In the BSE image, a thin brittle case was found to decorate the outer profile of the BMA sample (Figure 7b). The anodized layer contained some deep fine cracks (Figure 3) and had a flat fracture appearance. This shows that the secondary cracks grew in the direction normal to the external surface of the sample. Therefore, the cracking of the anodized layer resulted in degradation of the fatigue resistance of the anodized AA 7075-T6 alloy. Moreover, the presence of surface cracks within the anodized layer indicated that the crack-initiation stage should be unnecessary. Examining the fracture features around the anodizing layer at higher magnification, the fatigue crack propagated across the anodized coating and into the substrate, and strong bonding between the anodized coating and substrate was observed (Figure 7c). Moreover, a transgranular crack extended through the straight interface and showed the trace of the crack path (parallel stripes) just beneath the anodized layer (Figure 7d). This event confirmed the continuous progress of the crack without facing the barrier. It is obvious that strong bonding between the anodized layer and the substrate caused the fatigue crack growth rapidly through the interface (Figure 7c,d). In addition, a typical transgranular cleavage-like fracture was observed as the fatigue crack extended into the AA 7075 substrate (Figure 7d). Furthermore, a dimple fracture mixed with small facets was seen in the final fractured zone (not shown here).

**Figure 8:**
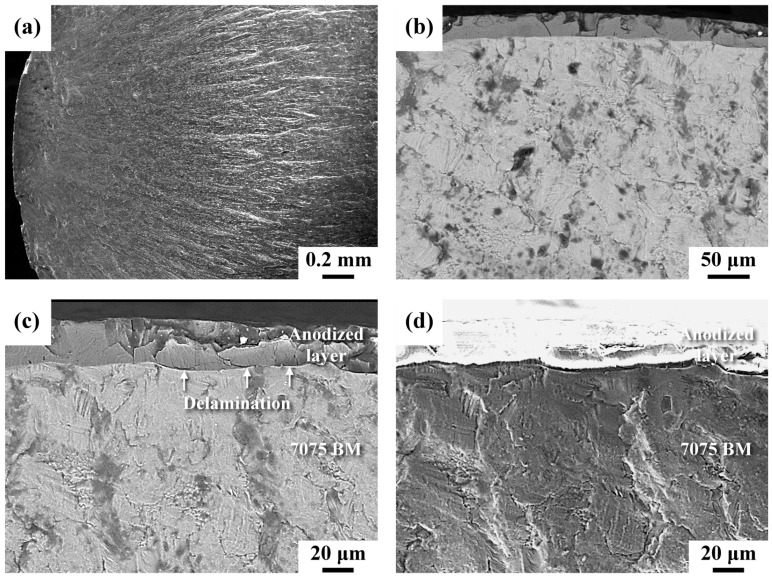
Fatigue fracture appearance of the HPA sample: (**a**,**d**) SE images; (**b**,**c**) BSE images. (**a**) Macro-fractured appearance of the crack initiation site; (**b**) enlarged view of the anodized layer; (**c**,**d**) fracture features around the anodized interface.

SEM photographs of the fractured appearance of the HPA specimen after fatigue tests are presented in Figure 8. The results indicated that the macro-appearance of the fractured HPA sample was similar to that of the BMA sample, consisting of a thin brittle case decorating the outer profile of the specimen (Figure 8a,b). It was noticed that the flat fracture of the anodized layer of the BMA sample was replaced by a chopped and smashed layer in the HPA sample (Figure 8c). Moreover, relatively large numbers of fine cracks and fragmental debris present in the anodized coating could be attributed to the brittle nature of the coating under high fatigue loading of the test. It was noticed that micro-shot peening before anodizing assisted the formation of a tortuous interface between the anodized layer and the AA 7075 substrate. Numerous microcracks assisted in dividing the anodized layer into many fine fragments after the fatigue test (Figure 8c). Moreover, the interface tended to delaminate as the crack growth passed it (indicated by the arrows in Figure 8c). In addition, a rubbed fracture feature was observed beneath the anodized layer (Figure 8d). It was noticed that the fatigue-fractured feature revealed a microscopic change in crack growth direction that occurred as the fatigue crack growth passed through the interface (Figure 8d). It was deduced that the interface separation seemed to deflect the crack growth direction (Figure 8d), which was beneficial in impeding the fatigue crack growth. The cleavage-like brittle fracture in the BMA sample was replaced by the squeezed rubbed feature in the HPA sample. Therefore, the CRS in the micro-shot peened specimen caused crack closure and had a great effect on retarding the fatigue crack growth of the anodized specimen.

**Figure 9:**
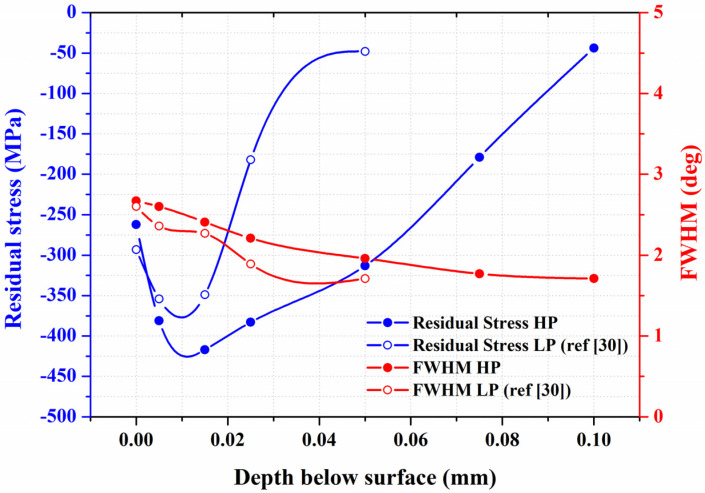
The changes in the residual stress and FWHM intensity in the X-ray diffraction pattern in the thickness direction of the LP and HP samples from the surface to the interior.

Figure 9 shows the changes in residual stress and FWHM intensity in the thickness direction of the LP [31] and HP samples from the surface to the interior. As mentioned previously, the increase in peening intensity caused an increase in surface roughness but was expected to increase the strength and depth of the residual stress field. Peak CRS was found in the subsurface zones of both peened samples. Moreover, the increase in peening intensity caused an obvious increase in depth of the CRS field. A steep decrease in residual stress to −48 MPa resulted in the LP specimen at a depth 50 μm below the peened surface. However, under the same CRS, the depth was increased to about 100 μm in the HP one. With the increase in peening intensity, the peak CRS increased from about −350 MPa to over −400 MPa, and the stress field also increased. The increased CRS field was definitely beneficial to the fatigue resistance to cracking. Moreover, the narrow CRS field meant that only a very limited deformed depth was introduced by the micro-shot peening.

## 4. Discussion

Although the anodized layer consisted of a few deep microcracks and fine defects of uneven sizes (Figure 3), the anodized layer was much harder (around HV 400) than the AA 7075-T6 substrate (around HV 180), as revealed in Figure 4. Thus, the anodized layer improved the resistance to corrosion and wear of AA 7075 alloy. The straight boundary between the anodized layer and the substrate of the unpeened specimen was replaced by a slightly tortuous trace of the peened one (Figure 3). It was noticed that the shallow dents with vague cracks were seen in the top surface of the micro-shot peened specimen. By contrast, scratches with rectangular microcracks were observed in the top surface of anodized unpeened specimen (Figure 3). Thus, the surface morphology and roughness of the anodized sample were affected by the surface texture of the original condition. In prior work, the micro-shot peened layer of AA 7075-T6 alloy comprised ultrafine grains in the outermost layer and micron-order elongated grains in the subsurface of the micro-shot peened specimen [31]. With increased peening intensity, the depth of the residual stress field and the surface roughness are expected to be increased due to deep deformation. The IPFs showed a nanogranular structure within a depth 10 μm below the anodized interface of the HPA sample (Figure 5a), but no such structure was detected in the LPA specimen (Figure 5b). It was clear that pickling before anodizing completely removed the fine-grained structure of the LPA sample. The retained fine structure, which was caused by the recrystallization of the heavily deformed zone during peening, was linked with the detectable strain within a depth of 20 μm beneath the anodized interface of the HPA sample (Figure 5c).

The results of fatigue tests in air revealed that the fatigue strengths/lives of the micro-shot peened (LP and HP) samples were much greater than that of the BM, particularly that of the LP one (Figure 6a). The high fluctuation in the fatigue strength/life of the HP sample in air, relative to that of the LP specimen, could be attributed to its inherent high surface roughness. Similar results have been reported: increasing the machined roughness of AA 7010-T7451 alloy from 0.6 to 3.2 μm caused a 32% drop in endurance limit in a low-stress state [11]. It was noted that hard anodizing had a very detrimental effect on the high-cycle fatigue of AA 7075 alloy shown in Figure 6b. The fatigue strength/life will be obviously shortened by the defects, especially those of surface defects [32]. A probabilistic fatigue life prediction model using the calibrated weakest-link theory has been proposed considering the superiority of the notch and surface defect [32,33]. With the presence of surface microcracks in the anodized coating, the fatigue limit of the BMA sample decreased to about 180 MPa. It was interesting that the fatigue strengths/lives of the peened anodized samples (LPA and HPA), especially that of the HPA, were much higher than that of the unpeened anodized (BMA) samples, as shown in Figure 6b. With micro-shot peening, the harmful effects of microcracks present in the anodized coating on the fatigue performance of AA 7075 alloy were mitigated. The difference in the outermost microstructure between the LPA and HPA samples was due to the retained nanocrystal structure of the latter (Figure 5a), which was hard to find in the former (Figure 5b). It was obvious that pickling almost completely removed the nano-grained structure of the LPA sample. It was deduced that precise control of the pickling process would highly alter the fatigue performance of anodized Al alloys, especially that of the micro-shot peened samples.

Micro-cracks initiate at corrosion pits around the surface and etch pits of AA 7050 alloy under cyclic loading [3]. Furthermore, fatigue failure of anodized Al alloy is often induced by the nucleation and growth of those micro-cracks formed in the anodic oxide film and through the interface into the substrate [9,10]. The stress concentration at the microcracks in the coating is responsible for the decreased fatigue properties of hard anodized 7475-T6 alloy [9]. It has been reported that pickling deteriorates the fatigue properties due to localized dissolution around the intermetallic compounds and/or inclusions [7,34,35], especially in the very-long-life regime [34]. Therefore, pickling was one of the controlling factors for the fatigue performance of the anodized Al alloys. As shown in Figure 7, fatigue cracks present in the brittle coating of an anodized sample grew through the coating/substrate interface and propagated directly into the substrate without the need of micro-crack initiation. This event illustrated the poor fatigue resistance of the BMA sample, as compared with that of the BM sample. Since micro-shot peening only influenced a thin external layer of the impacted material, the difference in the damaging mechanisms between the anodized samples with and without peening should only exist in the stage of retardation of crack growth below the anodized layer. The nano-grained structure and the CRS beneath the anodized layer were helpful in delaying or retarding fatigue crack propagation, which accounted for the higher fatigue strengths/lives of the peened anodized samples than that of the unpeened anodized one.

The macro-fractured appearances of the anodized unpeened and peened specimens were similar, as shown in Figure 7 and Figure 8. However, differences in the fracture features could still be distinguished between the samples in detail. Under high loading, the anodized layer of the peened sample was cracked into small patches (Figure 8c), in contrast to the overall flat fracture of the anodized layer of the unpeened sample (Figure 7c). Moreover, a change in the fatigue fracture appearance around the interface between the unpeened and micro-shot peened samples was observed. The crack propagated directly into the substrate of the unpeened sample, showing a cleavage-like fracture therein (Figure 7d). As the crack growth passed the interface of the micro-shot peened sample, delamination of the interface occurred (Figure 8c,d); this delamination could cause a change in the crack growth direction. It was noticed that a rubbed fracture feature instead of cleavage-like fracture was observed just below the anodized interface of the micro-shot peened sample (Figure 8d). The CRS around the anodized interface enhanced the crack closure, retarding the fatigue crack growth and leading to the rubbed fracture feature.

## 5. Conclusions

1. Micro-shot peening under two Almen intensities was performed to increase the fatigue strength/life of the anodized AA 7075-T6 alloy. Micro-cracks in the anodized layer significantly deteriorated the fatigue performance of AA 7075 alloy. Under high cycle fatigue, the endurance limit of the BMA sample was lowered to less than 200 MPa. Micro-shot peening could improve the fatigue strength/life of the anodized sample to the level of the unanodized substrate (about 300 MPa). Without anodizing, the fatigue performance of the HP sample was worse than that of the LP one. Moreover, fluctuation in the fatigue strength of the HP sample, relative to the LP sample, was attributed to the inferior effect of high surface roughness.
2. With an anodized layer, the fatigue strength of the HP sample was higher than that of the LP one. Pickling before anodizing eroded the outermost nanograins in the peened layer, which degraded the positive effect of micro-shot peening. Therefore, the HPA sample had higher fatigue resistance than the LPA one did. The fracture appearance of the anodized fatigued samples consisted of an observable ring of brittle fracture. Without any need for crack initiation, fatigue cracks present in the anodized layer propagated directly into the unpeened substrate, significantly reducing its fatigue strength/life. By contrast, the presence of CRS beneath the anodized layer of the micro-shot peened sample could retard the fatigue crack growth and was responsible for a noticeable increase in its fatigue performance. Moreover, interfacial separation between the anodized layer and peened surface could possibly deflect the crack path, which also contributed to the increased resistance to fatigue crack growth of the micro-shot peened sample.

## Figures and Tables

**Table 1:** The surface roughness values of the distinct specimens (unit: μm).

Specimen	Sa ^1^	Sp ^2^	Sv ^3^
Ground sample	0.217	1.494	−1.211
LP sample	0.385	1.396	−1.506
HP sample	1.303	6.400	−7.519

^1^ Sa—arithmetical mean height of the surface. ^2^ Sp—maximum peak height of the surface. ^3^ Sv—maximum pit depth of the surface.

## Data Availability

Data available on request due to restrictions eg privacy or ethical.
